# Systematic detection of m^6^A-modified transcripts at single-molecule and single-cell resolution

**DOI:** 10.1016/j.crmeth.2021.100061

**Published:** 2021-08-02

**Authors:** Kyung Lock Kim, Peter van Galen, Volker Hovestadt, Gilbert J. Rahme, Ekaterina N. Andreishcheva, Abhijeet Shinde, Elizabeth Gaskell, Daniel R. Jones, Efrat Shema, Bradley E. Bernstein

**Affiliations:** 1 Department of Pathology and Center for Cancer Research, Massachusetts General Hospital and Harvard Medical School, Boston, MA 02114, USA; 2 Broad Institute of MIT and Harvard, Cambridge, MA 02142, USA; 3 Department of Cancer Biology, Dana-Farber Cancer Institute, Boston, MA 02215, USA; 4 Division of Hematology, Brigham and Women's Hospital, Boston, MA 02115, USA; 5 Department of Pediatric Oncology, Dana-Farber Cancer Institute, Boston, MA 02215; 6 SeqLL Inc., Woburn, MA 01801, USA; 7 Department of Biological Regulation, Weizmann Institute of Science, Rehovot, Israel

**Keywords:** epitranscriptome, single molecule, single cell, multi-modal analysis, direct RNA sequencing, seqFISH, RNA modifications, m6A, epigenetics

## Abstract

Epigenetic modifications control the stability and translation of mRNA molecules. Here, we present a microscopy-based platform for quantifying modified RNA molecules and for relating the modification patterns to single-cell phenotypes. We directly capture mRNAs from cell lysates on oligo-dT-coated coverslips, then visually detect and sequence individual m^6^A-immunolabled transcripts without amplification. Integration of a nanoscale device enabled us to isolate single cells on the platform, and thereby relate single-cell m^6^A modification states to gene expression signatures and cell surface markers. Application of the platform to MUTZ3 leukemia cells revealed a marked reduction in cellular m^6^A levels as CD34^+^ leukemic progenitors differentiate to CD14^+^ myeloid cells. We then coupled single-molecule m^6^A detection with fluorescence *in situ* hybridization (FISH) to relate mRNA and m^6^A levels of individual genes to single-cell phenotypes. This single-cell multi-modal assay suite can empower investigations of RNA modifications in rare populations and single cells.

## Highlights

• Microscopy-based epitranscriptome profiling of low-input samples and single cells
• Single-molecule detection of m^6^A-modified RNA molecules
• Measurements of multiple modalities from the same single cells

## Motivation

Chemical modifications of mRNA affect diverse biological processes and are of crucial importance to cellular functions. However, the methodologies currently available to study these modifications are limited and incompatible with low-input samples or single cells. To overcome these limitations, we developed a microscopy-based platform to directly capture native mRNAs from cell lysates and detect m^6^A-modified RNA molecules at single-molecule resolution. Furthermore, we incorporated nanoscale technology to resolve single cells and thereby measure surface markers, gene expressions, and m^6^A levels on the same cells.

## Graphical abstract

**Figure:**
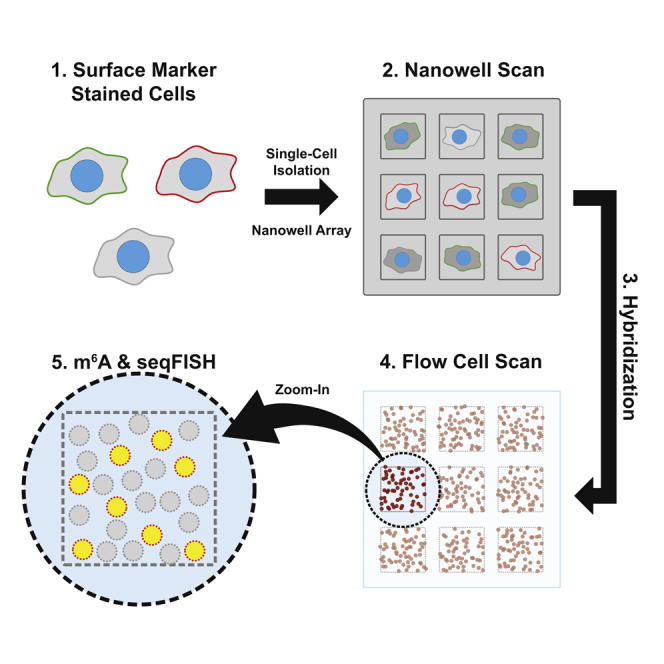

## Teaser

Kim et al. develop a microscopy-based platform for epitranscriptome profiling, compatible with low-input samples and single cells. The platform provides multi-modal measurements of cell surface markers, gene expression, and m^6^A RNA modification. The system is flexible and can be applied to study various RNA modifications and their dynamics in heterogeneous cell mixtures.

## Introduction

Chemical modifications of mRNA regulate transcript and protein abundance, thereby affecting cellular state. In addition to 5′ cap and 3′ polyadenylation, mRNAs can be modified via bases such as pseudouridine (Ψ), and methylation of adenosine and cytosine to produce *N*^*1*^-methyladenosine (m^1^A), *N*^*6*^-methyladenosine (m^6^A), and 5-methylcytosine (m5C) (Frye et al., 2018). The most abundant of these modifications is m^6^A, the levels of which vary widely between cell types and states, and 20%–40% of all mRNAs contain one or more m^6^A modifications (Dominissini et al., 2012; Frye et al., 2018; Meyer et al., 2012). m^6^A and its cognate writers, readers, and erasers have therefore emerged as essential regulators of gene expression (Yang et al., 2018).

Recent advances in transcriptome-wide m^6^A mapping technologies have broadened our understanding of m^6^A distribution and function (Dominissini et al., 2012; Meyer et al., 2012), but much is still out of reach. Antibody-based methods, such as m^6^A sequencing, MeRIP sequencing, and m^6^A-LAIC sequencing (m^6^A-LAIC-seq), provided the first transcriptome-wide view of m^6^A but require large amounts of input RNA (1∼3 μg) (Dominissini et al., 2012; Meyer et al., 2012; Molinie et al., 2016). Innovations in enzyme-based methods, such as MAZTER sequencing (Garcia-Campos et al., 2019) and DART sequencing (Meyer, 2019), reduce the RNA input requirement (10–100 ng) but are still limited to bulk samples and require complex library preparation. To build quantitative models of the impact of mRNA modifications on gene expression and cellular state, we require technologies that can quantify RNA modifications and transcript abundance—and ideally other measures of cellular state, such as surface markers—within single cells.

Toward this goal, we developed a microscopy-based platform to measure cell surface markers, gene expression, and m^6^A levels in individual cells and at single-molecule resolution. We combined innovations in nanowell technology (Gierahn et al., 2017), image registration, low-quantity digital gene expression (LQ-DGE) (Ozsolak et al., 2009), and sequential fluorescence *in situ* hybridization (seqFISH) (Eng et al., 2017) to generate data encompassing multiple parameters from single cells.

## Results

**Figure 1:**
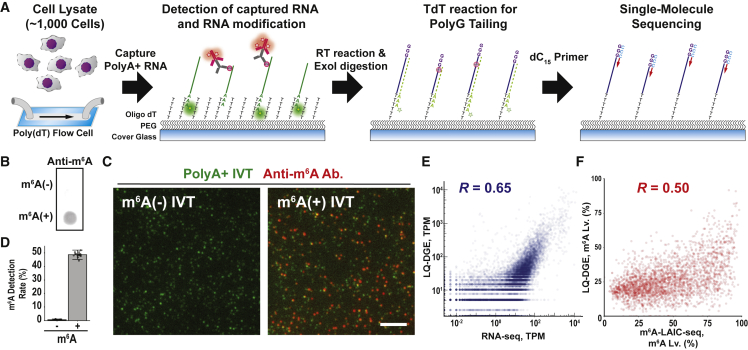
Transcriptome-wide m^6^A profiling at single-molecule resolution (A) LQ-DGE with m^6^A detection. PolyA^+^ RNA from ~1,000 GM12878 cells was captured on an oligo-dT-coated coverslip followed by antibody detection of m^6^A. Single-molecule sequencing of all transcripts was then performed by reverse transcription followed by second-strand cDNA synthesis. TdT, terminal deoxynucleotidyl transferase. (B) Dot blot assay with *in vitro* synthesized m^6^A^−/+^ transcripts (*in vitro* generated transcripts [IVTs]) using anti-m^6^A antibody. m^6^A^−^ IVTs were unmodified, and m^6^A^+^ IVTs contained an average of 12 m^6^ATP nucleotides per transcript. (C) TIRF microscopy images showing m^6^A^−^ or m^6^A^+^ Cy3-labeled IVTs (green) stained with an anti-m^6^A antibody and an Alexa Fluor 647-conjugated secondary antibody (red). Scale bar, 5 μm. (D) Quantification of m^6^A detection rates by analyzing colocalization of anti-m^6^A antibody and Cy3 fluorescence signals. (E) Scatterplot showing the correlation between modified LQ-DGE (0.51 M reads) and RNA sequencing (RNA-seq) (50 M reads) data for GM12878 cells. (F) Scatterplot showing the correlation between gene-specific m^6^A levels from the LQ-DGE (total, 0.51 M reads and m^6^A^+^, 0.14 M reads) and those from m^6^A-LAIC-seq (m^6^A-negative or m^6^A-positive sample, each 50 M reads). See also Figure S1 and Table S1.

We began by redesigning LQ-DGE technology, which combines sequential base additions with single-molecule total internal reflection fluorescence (TIRF) imaging (Ozsolak et al., 2009, 2010). We designed a surface with high antifouling performance to capture mRNA molecules (Figure 1A; STAR Methods). Briefly, we treated coverslips with azide-functionalized polyethylene glycol (PEG) to reduce non-specific binding of other biomolecules (Kim et al., 2018). We then coated the coverslips with alkyne-oligo-dT by copper-catalyzed azide-alkyne cycloaddition (“click reaction”). We used these surfaces to capture polyA^+^ RNA from cell extracts, which were then 3′-labeled with Cy3-dATP. We then used TIRF microscopy to register individual RNAs (Cy3 signal) and detect m^6^A-modified RNAs with a combination of m^6^A antibody and AF647-conjugated secondary antibody (Figure 1A). We extensively validated the sensitivity and linearity of our detection platform by using synthetic transcripts and 2× polyA^+^ RNA prepared from K562 cells deficient for either the m^6^A methyltransferase (*METTL3*-knockout [*METTL3*-KO]) or the cap-specific m^6^Am methyltransferase (*PCIF1*-KO) (Figures 1B–1E and S1; STAR Methods) (Boulias et al., 2019; Lin et al., 2016; Sendinc et al., 2019). Although the m^6^A antibody also recognizes the structurally similar *N*^6^, 2′-*O*-dimethyladenosine (m^6^Am) modification (Linder et al., 2015; Wei et al., 1975), our data indicate that this cap-specific modification contributes minimally to signal detected by our single-molecule assay in this system. Specifically, we find that *PCIF1*-KO leads to a minimal (∼5%) reduction in the fraction of 2× polyA^+^ RNA transcripts with detected m^6^A, in relation to wild-type (WT) K562 cells (Figures S1B and S1C). In contrast, *METTL3*-KO leads to a much more significant reduction (∼75%). The remaining 25% signal might reflect incomplete deletion of METTL3 in the cell line. Furthermore, liquid chromatography-tandem mass spectrometry (LC-MS/MS) analysis of absolute m^6^A and m^6^Am levels for 2× polyA+ RNA isolated from K562 (or YAC1) cells indicated that m^6^A is ∼20-fold more prevalent than m^6^Am, consistent with prior studies (Figure S1E) (Boulias et al., 2019; Molinie et al., 2016; Sendinc et al., 2019). The specificity of our assay for m^6^A-modified transcripts might reflect the fact that only 3′ polyA^+^ transcripts are captured and analyzed (Zhao et al., 2014), as well as the lower ratio of m^6^Am to m^6^A in our cell models.

Next, to identify modified and unmodified mRNA transcripts, we adapted single-molecule sequencing-by-synthesis methods (Ozsolak et al., 2009). We reverse transcribed the mRNA transcripts with oligo-dT primers to synthesize first-strand cDNA, digested excess primers with Exo I, and then used terminal transferase to append polyG tails to the 3′ ends of the cDNAs. We then sequenced the single molecules by using oligo dC_15_ primers and stepwise addition of fluorescent reversible terminator nucleotides (Figure 1A).

We first applied this procedure to mRNA isolated from 1,000 GM12878 cells, detecting m^6^A-modified and -unmodified mRNAs and sequencing corresponding cDNAs. We acquired a total of 0.5M sequencing reads, 27% of which were m^6^A modified (0.14M reads). This enabled us to directly quantify individual gene transcripts on the basis of mRNA counts, and to evaluate their m^6^A modification levels on the basis of the fraction that scored as m^6^A modified (Table S1). Biological replicates were highly concordant in terms of gene transcript levels (*R* = 0.97) and m^6^A-modified proportions (*R* = 0.92; Figures S1F–S1H). We also directly compared our data from 1,000 GM12878 cells against published data generated for 10 million GM12878 cells by using an m^6^A antibody immunoprecipitation (m^6^A-LAIC-seq) (Molinie et al., 2016). Despite the orthogonality of the assays, we found the datasets to be well correlated (whole transcriptome *R* = 0.65, m^6^A levels *R* = 0.50; Figures 1E, 1F, S1I, and S1J). Gene Ontology analysis of the data derived from our platform confirmed that transcriptional regulators are enriched among m^6^A-modified transcripts, whereas transcripts encoding translational regulators tend to have low levels of this modification (Molinie et al., 2016; Wang et al., 2019; Zhou et al., 2019) (Figure S1K). Altogether, these results demonstrate that our direct m^6^A detection and single-molecule sequencing can enable m^6^A profiling from very low numbers of cells with high specificity, sensitivity, and reproducibility.

**Figure 2:**
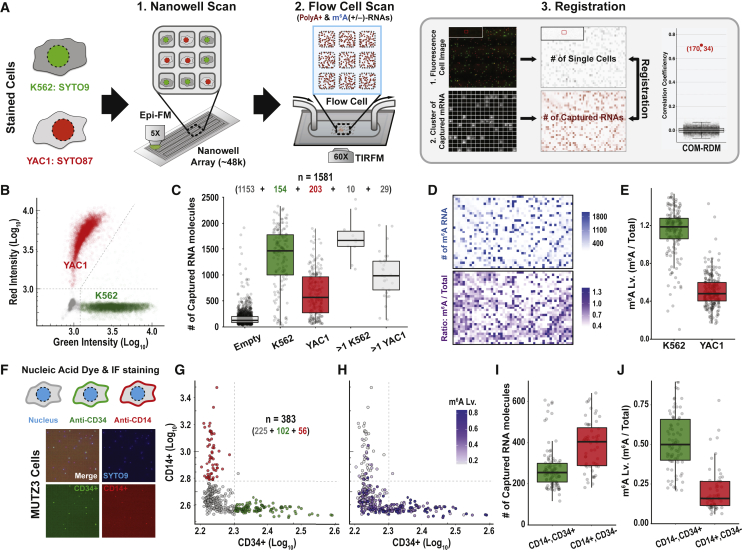
A platform for single-cell m^6^A level measurement (A) Workflow of m^6^A detection from single-cell transcriptomes. (1) Fluorescence dye-stained cells (K562, SYTO9 [green]; YAC1, SYTO87 [red]) were allowed to settle into the wells of a nanowell plate by gravity. The plate was scanned by epifluorescence microscopy to count and phenotype cells prior to lysis. (2) After cell lysis, the nanowell plate was immediately sealed with an oligo-dT-coated coverslip to capture polyA^+^ RNAs confined in each well. A flow cell was assembled with the detached coverslip. Captured polyA^+^ RNA molecules were labeled with Cy3-dATP by using Klenow exo-. m^6^A-modified RNA molecules were detected as described in Figure 1A. The flow cell was scanned by using TIRF microscopy to measure the amounts of captured polyA^+^ RNA and m^6^A-modified RNA molecules. (3) The nanowell and flow cell scanning images were used to generate a COM and single-molecule density matrix of captured RNA (RDM), respectively. Occupied wells were registered by finding the outlier of correlation coefficient between the matrices. (B) Scatterplot of single-cell fluorescence intensity from nanowell scanning images with K562 (green) and YAC1 (red) cells. (C) Box plots depicting the number of captured polyA^+^ RNA molecules per imaging area (15,400 μm^2^). Number of imaging areas is indicated above box plots. (D) Matrices of single-molecule density of m^6^A-modified polyA^+^ RNA (top) and the ratio of m^6^A-modified polyA^+^ RNA to total polyA^+^ RNA molecules (bottom). (E) Box plots showing m^6^A levels for K562 and YAC cells. (F) Representative fluorescence images of nanowells occupied with MUTZ3 cells stained with SYTO9 (blue), anti-CD14 (red), and anti-CD34 (green). (G and H) Scatterplot of single-cell fluorescence intensity (G) from nanowell scanning images of stained MUTZ3 cells. Each cell was colored by corresponding m^6^A level (H). (I and J) Box plots showing the number of captured polyA^+^ RNA molecules (I) and m^6^A levels (J) in CD14^−^CD34^+^ and CD14^+^CD34^−^ cell populations. See also Figure S2.

We next extended our platform to quantify transcripts and m^6^A levels for single cells (Figure 2A). We designed an array with 47,368 subnanoliter wells (0.6 nL per well). We reasoned that the physical isolation of single cells into each nanowell would facilitate imaging of multiple modalities from the same single cell, both before and after cell lysis. We loaded a mixture of K562 (human) and YAC1 (mouse) cells stained with SYTO9 and SYTO87, respectively, into the wells by using gravity. The cell preparation was diluted such that most wells were loaded with a single cell. We scanned the loaded array with multicolor fluorescence imaging (5× magnification) to enable counting and phenotyping of cells prior to lysis (Figure 2A, Nanowell Scan). We then converted the x/y coordinates of each nanowell along with the number of loaded cells from the fluorescence image into a cell occupancy matrix (COM) for the array. This step facilitates cell phenotyping without sorting (Figure 2B), and acts as a quality control by measuring cell density in the array.

Next, we added lysis solution to the nanowells, and sealed the array with an oligo-dT-coated coverslip with the surface chemistry described above (Figures S2A and S2B). We incubated the assembly to capture polyA^+^ RNAs, and assembled a flow cell (Figure 2A; STAR Methods). We detected bound RNA molecules and m^6^A by imaging each surface area of 15,400 μm^2^ for individual nanowells using TIRF microscopy (60× magnification), as detailed above and in Figure 1 (STAR Methods). The images revealed a grid-like pattern with multiple squares with high RNA signal density separated by thin frames without signal. Alignment of the TIRF image to the lower-resolution fluorescent scan allowed us to register the TIRF squares to 1,581 individual nanowells (Figures S2C and S2D). We then implemented a custom algorithm to quantify polyA^+^ RNAs and m^6^A in each nanowell and converted this information into an RNA density matrix (RDM) of transcript abundance and m^6^A modification levels for individual cells (Figure 2A, Flow Cell Scan). Nanowells with zero cells or with more than one cell were excluded from further analysis.

The matrix registration between COM and RDM enabled us to link the cellular phenotype (green/K562 and red/YAC1, Figure S2E) to the molecular readouts for each single cell. We found that K562 cells contain ∼2-fold greater transcript abundance and ∼2-fold higher m^6^A levels compared with YAC1 cells (Figures 2C–2E). Similar trends of higher m^6^A levels in K562 cells were confirmed by traditional dot blot assay (Figure S2F) and high-sensitivity mass spectrometry (LC-MS/MS; Figure S1E). We conclude that the nanowell version of our platform enables quantification of m^6^A-modified RNAs and multicolor phenotyping of the same sample on a single-cell level.

To demonstrate the utility of our platform for measuring immunophenotypes, we cultured human acute myeloid leukemia cells (MUTZ3) in media conditions that induced partial differentiation. We then incubated the cells with a nuclear stain (SYTO9) and antibodies against CD34, a surface marker of leukemic progenitors, and CD14, a marker of myeloid differentiation (Vu et al., 2017; Weng et al., 2018) (Figures 2F and 2G). We loaded labeled MUTZ3 cells into the nanowell array and used our platform to assign an immunophenotype, as well as quantifying total polyA^+^ RNAs and m^6^A-modified RNAs in each single cell (Figures 2H–2J). Similar to previous reports on bulk populations of cells, we find that primitive CD34^+^, CD14^−^ MUTZ3 cells contain 40% less polyA^+^ RNAs but 3 times more m^6^A-modified RNAs than differentiated CD34^−^, CD14^+^ cells (Vu et al., 2017; Weng et al., 2018). Again, we confirmed m^6^A levels by fluorescence-activated cell sorting (FACS) using CD14/CD34 gates and by m^6^A dot blot (Figure S2G). These data demonstrate that our platform can jointly assign an immunophenotype and quantify m^6^A-modified RNA transcripts in the same single cells, without cell sorting.

**Figure 3:**
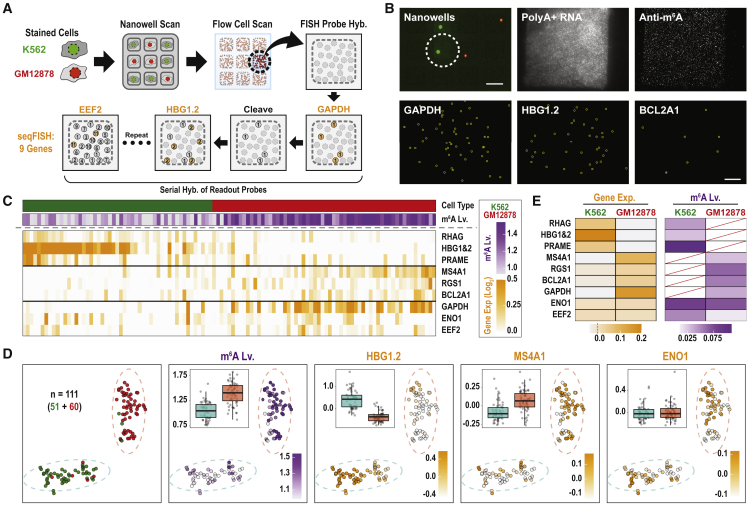
Application of seqFISH on the single-cell m^6^A assay (A) Experimental workflow. A mixture of K562 (SYTO9, green) and GM12878 (SYTO87, red) cells was applied to the nanowell plate. The nanowell plate and flow cell were scanned, as described in Figure 2A. Gene-specific primary probes were hybridized to nine targeted mRNAs. To quantify each targeted transcript, we sequentially hybridized secondary readout probes, removing the fluorophores between rounds by disulfide cleavage. (B) Top left: representative nanowell scan. Scale bar, 100 μm. Remaining images show polyA+ RNA; m^6^A-modified RNA; and seqFISH for GAPDH, HBG1 and 2, and BCL2A1 from a single well. Scale bar, 20 μm. (C) Heatmap showing m^6^A levels and relative expression levels of nine target genes at a single-cell level in K562 (green) and GM12878 (red) cells. (D) tSNE visualization of 48 qualified single-cell seqFISH profiles. Dashed circles indicate clusters (subpopulations). Single cells are colored according to cell type on the nanowell array (green or red), m^6^A level (purple), or relative gene expression levels (orange). Box plots show the m^6^A level (m^6^A/total transcripts) or log-mean gene expression levels among clusters. (E) Heatmap of gene expression levels and gene-specific m^6^A levels in K562 and GM12878 cells. Red diagonal lines represent “not applicable” values with log-mean gene expression levels less than −0.02. See also Figure S3 and Table S2.

We next sought to apply the platform to quantify surface markers, m^6^A levels, and gene expression signatures in the same single cells (Figure 3). To quantify transcripts on our single-molecule surfaces, we implemented a seqFISH (Eng et al., 2017). We applied a mixture of pre-stained K562 (SYTO9; green) and GM12878 (SYTO87; red) onto the nanowell array (Figures 3A and 3B). We proceeded through the steps detailed above to query surface marker expression, to lyse the cells, to quantify polyA^+^ RNAs, and to quantify m^6^A-modified RNAs in each single cell. Next, we hybridized an OligoPool of 222 primary probes targeting the coding regions of 9 mRNAs (18–30 probes per gene) to the flow cell. To quantify absolute counts for each targeted transcript, we sequentially hybridized secondary probes, removing the fluorophores between rounds by disulfide cleavage (Figures 3A and 3B and Table S2).

We acquired high-quality surface marker data, m^6^A levels, and gene expression signatures for 111 single cells. We performed unsupervised clustering on the gene expression signatures to distinguish cells types, validating the clusters by surface marker status. The seqFISH data enabled us to confidently assign each cell as K562 or GM12878 (Figures 3C, 3D, and S3A). This allowed us to compare m^6^A levels at the single-cell level, which revealed higher transcript abundance but lower m^6^A levels in the K562 cells, consistent with our initial bulk analysis (Figures S3B and S3D).

Finally, we sought to couple m^6^A detection and seqFISH to quantify m^6^A levels on known transcripts in single cells (Figures 3E and S3E). In the 36 single cells with successful image alignment between m^6^A detection and seqFISH, we found that 436 out of 8,837 transcripts for 9 genes were modified. Quantification of m^6^A levels at the resolution of single genes is well correlated between our bulk and single-cell experiments (*R* = 0.48; Figure S3F). It is worth noting that, because of the nature of single-cell data, reproducible measurements of m^6^A on single transcripts from single cells are only feasible in transcripts expressed over a certain threshold (Figures S3G–S3I; STAR methods). Altogether, these experiments provide a proof of principle that our platform is able to profile cell surface markers, and to quantify both transcripts and their m^6^A levels, all from the same single cell.

## Discussion

In summary, we have established an open source platform for multi-modal single-cell assays. At the bulk level, we demonstrated transcriptome-wide profiling and quantification of RNA modifications from low-input samples (<1,000 cells). At the single-cell level, the nanowell adaptation enabled simultaneous quantification of cell surface markers, total polyA^+^ RNA content, RNA modifications, and absolute numbers of individual transcripts, from the same single cells. Direct imaging and image registration between the cellular phenotyping and molecular imaging steps abrogates the need for cellular barcodes, obviating laborious library preparation steps. The open source nature and flexibility of our platform also lends itself to the future addition of other modalities, such as antibody probes (Figures S3J–S3K) and chemical detection of other RNA modifications (Li et al., 2015). This could enable investigation of combinatorial RNA modifications (“code”) or charting biophysical properties of RNA-binding proteins (Meyer, 2019). We expect that future versions of the nanowell technology will enable RNA density optimization, and further integration with single-molecule sequencing methods (i.e., LQ-DGE) will increase the transcriptome-wide throughput and enable the measurements of individual isoforms and allelic expression (Wang et al., 2009). In conclusion, we present an effective and flexible platform for the measurement of epigenetic modification of mRNAs, transcript abundance, and surface proteins at the single-molecule and single-cell levels.

### Limitations

Our current procedure for detecting RNA modifications on single molecules favors binary interpretation of the presence or absence of the modification over stoichiometry of the modification sites in a transcript. Regardless of the number of m^6^A-modified sites in a transcript, the fluorescence signal from antibody detections is converted to m^6^A-positive transcripts during processing of the single-molecule image. As a result, our assay classifies transcripts as unmodified or as containing one or more modifications. Going forward, quantitative measurements of modification sites in a transcript might be accomplished by labeling modified bases enzymatically with fluorescence dye (Shu et al., 2020; Wang et al., 2020) or fluorescently tagged modification-specific RNA-binding protein (Ries et al., 2019), followed by stoichiometric measurements by single-molecule photobleaching (Ulbrich and Isacoff, 2007). An additional issue relates to cross-reactivity of the m^6^A antibody with the cap-specific m^6^Am modification (Linder et al., 2015). Although our controls suggest that m^6^Am contributes minimally to the detected signal in our experimental system (Figures S1B–S1E), this cross-reactivity could confound interpretation of data from other systems. Therefore, we recommend complementing analysis of the single-cell data with assays such as m^6^Am-exo sequencing (Boulias et al., 2019), DART sequencing (Meyer, 2019), and/or liquid chromatography-mass spectrometry (LC-MS) analysis.

## STAR★Methods

### Key resources table

**Table:** 

Reagent or Resource	Source	Identifier
**Antibodies**		

Rabbit, Anti-N6-methyladenosine antibody	Cell Signaling Technology	Cat# 15000BC
Anti-rabbit IgG, HRP-linked antibody	Cell Signaling Technology	Cat# 7074S; RRID: AB_2099233
Mouse, Anti-CD14 antibody, RMO52	Beckman Coulter	Cat# IM0643
Monoclonal mouse, Anti-CD34 antibody, clone 8G12	BD Biosciences	Cat# 348050; RRID: AB_400369
Goat anti-Mouse IgG1 Cross-Adsorbed Secondary Antibody, Alexa Fluor 546	ThermoFisher	Cat# A-21123; RRID: AB_2535765
Goat anti-Mouse IgG2a Cross-Adsorbed Secondary Antibody, Alexa Fluor 647	ThermoFisher	Cat# A-21241; RRID: AB_2535810
Goat anti-Rabbit IgG (H+L) Cross-Adsorbed Secondary Antibody, APC	ThermoFisher	Cat# A-10931; RRID: AB_2534068
Rabbit anti-PCIF1 Antibody	Bethyl Laboratories	Cat# A304-711A; RRID: AB_2620906
Rabbit anti-METTL3/MT-A70 Antibody	Bethyl Laboratories	Cat# A301-567A; RRID: AB_1040004

**Chemicals, peptides, and recombinant proteins**		

3-(2-aminoethylamino)-propyltrimethoxysilane	Tokyo Chemical Industry Co.	Cat# A0774
Acetic acid	Sigma	Cat# A6283
Azide PEG Succinimidyl Carboxymethyl Ester	JenKem Technology USA Inc.	Cat# A5088-1
Dimethyl sulfoxide, anhydrous, ≥99.9%	Sigma	Cat# 276855
Triethylamine	Sigma	Cat# 471283
Copper(II) sulfate pentahydrate	Sigma	Cat# 209198
Tris-hydroxypropyltriazolylmethylamine (THPTA)	Click Chemistry Tools	Cat# 1010-100
Sodium ascorbate	Sigma	Cat# A4034
N6-methyladenosine-5’-triphosphate	TriLink	Cat# N1013
1 M MgCl2	Sigma	Cat# 63069-100ML
1 M Tris-HCl pH 8.0	Boston BioProducts	Cat# BBT-80
10 mM dNTPs	New England BioLabs	Cat# N0447L
BSA	Sigma	Cat# A9418-100G
RNase Inhibitor	Thermo Fisher	Cat# AM2696
Tween-20	Fisher Scientific	Cat# 65-520-4100ML
UltraPure™ 0.5M EDTA, pH 8.0	Invitrogen	Cat# 15575020
Sodium Chloride	Fisher Chemical	Cat# S671-3
Potassium hydroxide	Sigma	Cat# P5958
10% NP-40	Abcam	Cat# ab142227
pCp-Cy3	Jena Bioscience	Cat# NU-1706-CY3
Protocatechuate 3,4-Dioxygenase	Sigma	Cat# P8279-25UN
Protocatechuic acid	Sigma	Cat# P5630
Trolox	Sigma	Cat# 238813
Amersham Hybond-XL	Cytiva	Cat# RPN303s
Cyanine 3-dATP	PerkinElmer	Cat# NEL592001EA
1X ThermoPol buffer	New England BioLabs	Cat# B9004S
Virtual terminator dNTP analogs	SeqLL	N/A
Adenosine	Toronto Research Chemicals	Cat# A280400
2'-Deoxyadenosine Monohydrate	Toronto Research Chemicals	Cat# D231620
N6-Methyladenosine	Toronto Research Chemicals	Cat# M275895
N6,O2'-Dimethyladenosine	Toronto Research Chemicals	Cat# D447415
Hoechst 33342	Invitrogen	Cat# 62249
SYTO9	Invitrogen	Cat# S34854
SYTO87	Invitrogen	Cat# S11363
Hybridization Chamber Kit - SureHyb enabled	Agilent	Cat# G2534A
FluoSphere beads	Thermo Fischer	Cat# F8807
Azido-PEG3-SS-NHS	Conju Probe	Cat# CP-2060
AFDye 647 DBCO	Click Chemistry Tools	Cat# 1302-1
Azide Magnetic Beads	Click Chemistry Tools	Cat# 1036-1
20X SSC	Thermo Fischer	Cat# 15557036
Formamide	Sigma	Cat# F9037
Dextran Sulfate	Sigma	Cat# D8906; D4911
TCEP	Sigma	Cat# 646547

**Critical commercial assays**		

HiScribe™ T7 Quick High Yield RNA Synthesis Kit	New England BioLabs	Cat# E2050
RNAClean XP Kit	Beckman Coulter	Cat# A66514
*E*. *coli* Poly(A) Polymerase	New England BioLabs	Cat# M0276
T4 RNA Ligase 1	New England BioLabs	Cat# M0204
Dynabeads mRNA DIRECT Purification Kit	Invitrogen	Cat# 61011
Pierce™ ECL Western Blotting Substrate	Thermo Fisher	Cat# 32106
RNeasy Mini Kit	QIAGEN	Cat# 74104
Klenow Fragment (3’→5’ exo-)	New England BioLabs	Cat# M0212S
Proteinase K	New England BioLabs	Cat# P8107S
SuperScript III Reverse Transcriptase	Invitrogen	Cat# 18080093
Exonuclease I	New England BioLabs	Cat# M0293S
Terminal Transferase	New England BioLabs	Cat# M0315S
RppH	New England BioLabs	Cat# M0356S
RNA Clean and Concentrator-5 Kit	Zymo Research	Cat# R1013
Nuclease P1	New England BioLabs	Cat# M0660
Shrimp Alkaline Phosphatase	New England BioLabs	Cat# M3071S
YM-10 centrifugal spin column	Millipore	Cat# MRCPRT010
Illustra NAP-5 columns	GE Healthcare	Cat# 17-0853-01

Deposited Data		

m^6^A-LAIC-seq	Molinie et al., 2016	GSE66086

**Experimental models: Cell lines**		

K562	ATCC	Cat# CCL-243
YAC1	ATCC	Cat# TIB-160
GM12878	Coriell	Cat# GM12878
MUTZ3	DSMZ	Cat# ACC-295
5637	ATCC	Cat# HTB-9

**Oligonucleotides**		

*in vitro*-generated transcripts (IVTs)	Integrated DNA Technologies	5’-GGCCAGTGAATTGTAATACGACTC ACTATAGGGAGGCGGTAACACCTTC TGGACTCTTCATAGAGTTGGTCTATT TGTCTCCACGCTGCCAGGTTGTTGT GGCCTGTTTTCGGGCGTCTGCGGC GAAGATCTCTTGTCAGAGCCTTAGG TGTATCTAGATTGTGATCCCCTTTCC TCACTTGGTAGTCTGTCGACTT-3’
Hexynyl-Oligo dT50	Integrated DNA Technologies	5’Hexynyl-TTTTTTTTTTTTTTTTTTTTT TTTTTTTTTTTTTTTTTTTTTTTTTTTTT-3’
Synthetic RNA fragments	Integrated DNA Technologies	rArC-rX-rUrG, X=A, m6A, U or ψ
METTL3 gRNA_1	Integrated DNA Technologies	5’-ATCATTCGGACAGGCCGTAC-3’
METTL3 gRNA_2	Integrated DNA Technologies	5’-GCTCAACATACCCGTACTAC-3’
PCIF1 gRNA_1	Integrated DNA Technologies	5’-GATCCGTTTGACGTACTCCA -3’
PCIF1 gRNA_2	Integrated DNA Technologies	5’-ACTTAACATATCCTGCGGGG-3’
Control gRNA_1	Integrated DNA Technologies	5’-ACGGAGGCTAAGCGTCGCAA-3’
Control gRNA_2	Integrated DNA Technologies	5’-CGCTTCCGCGGCCCGTTCAA-3’
seqFISH probes	Integrated DNA Technologies	See the Table S2

**Recombinant DNA**		

pGIR-PB-U6-NT1(H.s)-CMV-Cas9-T2a-eGFP	Table S3	N/A
pGIR-PB-U6-NT2(H.s)-CMV-Cas9-T2a-eGFP	Table S3	N/A
pGIR-PB-U6-METTL3-gRNA1-CMV-Cas9-T2a-eGFP	Table S3	N/A
pGIR-PB-U6-METTL3-gRNA2-CMV-Cas9-T2a-eGFP	Table S3	N/A
pGIR-PB-U6-PCIF1-gRNA1-CMV-Cas9-T2a-eGFP	Table S3	N/A
pGIR-PB-U6-PCIF1-gRNA2-CMV-Cas9-T2a-eGFP	Table S3	N/A

**Software and algorithms**		

Scripts used for image processing and single-cell analysis	This paper	https://github.com/Kim-KL/scRNAmod
R version 3.4	R Core Team	https://www.r-project.org
ImageJ 1.46 r	NIH	https://imagej.nih.gov/ij/

### Resource availability

#### Lead contact

Further information and requests for resources and reagents should be directed to and will be fulfilled by the Lead Contact, Bradley E. Bernstein (Bradley_Bernstein@DFCI.HARVARD.EDU).

#### Materials availability

Plasmids generated in this study are available upon request.

#### Data and code availability

The custom scripts generated during this study are available on GitHub (https://github.com/Kim-KL/scRNAmod).

### Experimental model and subject details

K562 and YAC1 cells (ATCC) were cultured using RPMI 1640 medium (Gibco, Cat. #61870036) supplemented with 10% heat-inactivated fetal bovine serum (FBS). GM12878 cells were cultured using RPMI 1640 medium (Gibco, Cat. #61870036) supplemented with GlutaMax and 15% FBS. MUTZ3 cells were cultured using MEM alpha (Thermo, Cat. #12571-063) with 20% FBS and 10% 5637-conditioned medium. All cells were maintained at 37°C in a humidified CO_2_-controlled (5%) incubator.

5637 cells were cultured using RPMI 1640 medium supplemented with 10% FBS until 95% confluence. The culture medium was collected from the culture dish and centrifuged for 10 minutes at 1,200 rpm and filtered through a 0.2-μm Millipore filter. This conditioned medium retained its potency for a couple of weeks when stored at 4°C or for several months when stored at -20°C.

### Method details

#### Preparation of oligo dT surface

Coverslips were functionalized as previously described (Kim et al., 2018) with some modifications. Briefly, extensively cleaned coverslips were prepared by washing with ultrapure water and 1 M KOH for at least 2 h, and then were treated with 1.5% 3-(2-aminoethylamino)-propyltrimethoxysilane (Tokyo Chemical Industry Co., Cat. #A0774) in ethanol solution with 5% (*v/v*) acetic acid for 20 min at room temperature. After triple rinsing with ethanol, the coverslips were dried and doped with 100 mg/mL azide-PEG (JemKem Technology USA, Cat. #A5088-1) in anhydrous DMSO solution with 0.1% (*v/v*) triethylamine for at least 2 h at room temperature. After extensive rinsing with ultrapure water, the passivated coverslips were dried and stored in a vacuum desiccator until oligo deposition. The coverslips were doped with Hexynyl-Oligo dT50 in 0.1 M sodium bicarbonate buffer with 2 mM CuSO_4_, 2mM THPTA, and 4 mM sodium ascorbate for 1 h at room temperature and then extensively washed with ultrapure water and kept in T50 buffer (10 mM Tris-HCl pH 8.0, 50 mM NaCl, 0.05% Tween-20) until the next step.

#### Anti-m^6^A antibody validation

To validate the anti-m^6^A antibody, we used *in vitro*-generated transcripts (IVTs) from a DNA oligo of random sequence: (T7 promoter) 5’-GGCCAGTGAATTGTAATACGACTC-ACTATAGGGAGGCGGTAACACCTTCTGGACTCTTCATAGAGTTGGTCTATTTGTCTCCACGCTGCCAGGTTGTTGTGGCCTGTTTTCGGGCGTCTGCGGCGAAGATCTCTTGTCAGAGCCTTAGGTGTATCTAGATTGTGATCCCCTTTCCTCACTTGGTAGTCTGTCGACTT-3’). The DNA oligo was ordered from Integrated DNA Technologies and *in vitro* T7-mediated transcription was performed using the HiScribe™ T7 Quick High Yield RNA Synthesis Kit (NEB, Cat. #E2050) as described in the user manual using 0% or 50% N6-methyladenosine-5’-triphosphate (TriLink, Cat. #N1013) during synthesis. After the purification of IVTs using the RNAClean XP Kit (Beckman Coulter, Cat. #A66514), a poly(A) tail was added to the end of IVTs using *E*. *coli* Poly(A) Polymerase (NEB, Cat. #M0276) as described in the user manual using 100% Adenosine-5’-Triphosphate. IVTs were labeled with pCp-Cy3 (Jena Bioscience, Cat. #NU-1706-CY3) on the end of the poly(A) tail using T4 RNA Ligase 1 (NEB, Cat. #M0204).

A custom Secure Seal Flowcell was built with the passivated poly(dT50) coverslips. For antibody validation, 0.2 ng of IVT in T50 buffer with 20 U SUPERase·In RNase Inhibitor (Ambion, Cat. #AM2694) were hybridized to the poly(dT50) surface at room temperature for 1 h. Next, to label m^6^A-positive IVTs, the pre-complex of 67 pM anti-m^6^A primary antibody and 200 pM APC-labeled secondary antibody in Imaging buffer (10 mM Tris-HCl pH 8.0, 50 mM NaCl, 0.05% Tween-20, 1 mM Trolox, 50 nM Protocatechuate 3,4-Dioxygenase, 1 mg/mL protocatechuic acid, 0.5 mg/mL bovine serum albumin, 20 U SUPERase·In RNase Inhibitor) was applied to the flow cell and bound for 15 min at 37°C. After incubation, the imaging cycle was repeated 6 times using customized total internal reflection fluorescence (TIRF) microscopy (Nikon TE with custom-built laser assembly, a Nikon 60X oil objective, and Photometrics CoolSNAP HQ CCD camera). In the two-color images of the same region using Cy3 as an IVT and APC as an antibody complex, respectively, the fluorescent spots were fitted with Gaussian profiles to determine the center positions of the molecules to half-pixel accuracy. The fluorescent spots between two color images, whose center was within a distance of two pixels (∼200 nm), were determined as colocalization spots. The detection rate was measured by the colocalization rate of antibody complex and IVT. The antibody sensitivity was measured as the detection rate of true positive on an m^6^A-positive IVT sample and the specificity as the detection rate of true negative on an m^6^A-negative IVT sample.

#### Dot blot assay for RNA modifications

PolyA+ RNA selection was performed twice using Dynabeads mRNA DIRECT Purification Kit (Invitrogen, Cat. #61011) as described in the user manual. 2x polyA+ RNA samples were spotted onto the membrane, Amersham Hybond-XL (Cytiva, Cat# RPN303s). The membrane was completely dried and crosslinked in a UV STRATALINKER 1800 using the automatic function. The membrane was then blocked for 10 min using sterile RNase, DNase-free TBST + 5% skim milk. The m^6^A primary antibody was then added at a concentration of 1:1,000 in TBST + 5% skim milk at 4°C, overnight. The membrane was washed four times in TBST and then incubated with the secondary anti-rabbit antibody (1:5,000) for 1 h in TBST + 5% skim milk. The membrane was washed four times in TBST and exposed on the ChemiDoc imaging system (Bio-Rad) using Pierce ECL Western Blotting substrate.

#### Direct RNA sequencing with detection of m^6^A

The total RNA of GM12878 cells was extracted using the RNeasy Mini Kit (QIAGEN, Cat. #74104). 1 ng total RNA in T50 buffer or cell lysates from 1,000 cells in lysis buffer (20 mM Tris-HCl, pH 7.4, 150 mM NaCl, 1 mM MgCl_2_, 1 mM EDTA, 0.5% NP-40, 20 U SUPERase·In RNase Inhibitor) was directly captured on the passivated poly(dT50) flow cell for 30 min at room temperature. After rinsing with 1X SSC/ 0.05% SDS three times, the captured RNA was labeled with 100 nM Cy3-dATP using Klenow exo- (NEB, Cat. #M0212) according to the manufacturer’s instructions and was incubated with the pre-complex of anti-m^6^A antibody in the imaging buffer for 15 min at 37°C, as described above for antibody validation. The imaging cycle was repeated 6 times using a customized TIRF microscopy system, and the flow cell was cleared of antibody complex by Proteinase K in the T50 buffer. After rinsing with the T50 buffer three times, first-strand cDNA was synthesized on the flow cell as previously described with some modifications (Ozsolak et al., 2010). First-strand cDNA was synthesized with the SuperScript III Reverse Transcriptase (Invitrogen, Cat. #18080093) using the manufacturer’s recommendations, except no additional primers were added, and the incubation steps were modified as follows: 37°C for 15 min and 55°C for 15 min. After cDNA synthesis, the unoccupied dT oligos were degraded using Exonuclease I (NEB, Cat. #M0293), and simultaneous poly(G) tailing and RNA degradation were performed using Terminal Transferase (NEB, Cat. #M0315S) as described in the user manual adding 1 mM dGTP and RNase H. After incubation at 37°C for 15 min, 3’ ends of poly(G) tails were blocked with a mixture of 0.1 mM ddGTP and 0.1 mM ddATP under the same reaction conditions. The 15-nt poly(dC) primers were hybridized at 50 nM in T50 buffer at 37°C for 15 min, followed by step-wise ‘fill’ steps with Klenow exo- with a mixture of 0.5 mM dCTP and 0.5 mM dATP according to the manufacturer’s instructions. Then, the ‘lock’ step was performed with Virtual Terminator guanine and Virtual Terminator thymidine nucleotide analogs (SeqLL). Sequencing by synthesis was then initiated using standard procedures (Ozsolak et al., 2010).

#### m^6^A level calculation

m^6^A level of a specific gene was calculated as a percentage of modified gene-specific transcripts to the total gene-specific transcripts. We required total gene-specific transcript counts of ≥ 10 or log-mean gene expression levels of ≥ 0.02 to obtain reliable m^6^A levels. Single-cell m^6^A levels were calculated using total RNA counts and m^6^A modification counts in each nanowell.

#### Identification of multiple RNA modifications

To test iterative detection of multiple RNA modifications, we used anti-m^6^A and pseudouridine (*Ψ*) antibodies. To validate antibody specificity, the synthetic RNA fragments (rArC-rX-rUrG, X = A, m^6^A, U or *Ψ*) were ordered from Integrated DNA Technologies. Synthetic RNA fragments (50 ng) were spotted onto the charged nylon membrane, and the antibodies were applied to the membrane with a dot blot assay. Cell lysates in lysis buffer (20 mM Tris-HCl, pH 7.4, 150 mM NaCl, 1 mM MgCl_2_, 1 mM EDTA, 0.5% NP-40, 20 U SUPERase·In RNase Inhibitor) were directly captured on a poly(dT50) flow cell for 30 min at room temperature. After rinsing with 1X SSC/ 0.05% SDS three times, the captured RNA was labeled with Cy3-dATP by Klenow exo- and was incubated with the pre-complex of anti-m^6^A antibody in imaging buffer for 15 min at 37°C. Imaging for m^6^A-modified RNAs was done using customized TIRF microscopy, and then the flow cell was cleared of antibody complex by 40 U/mL Proteinase K in T50 buffer. After rinsing three times with T50 buffer, the imaging process was repeated with the anti-*Ψ* antibody.

#### Generation of *METTL3* or *PCIF1* knockout cell lines

K562 *METTL3*- or *PCIF1*-knockout cell lines were generated by CRISPR/Cas9 using piggybac vectors. The gRNAs used were: *METTL3*: ATCATTCGGACAGGCCGTAC or GCTCAACATACCCGTACTAC; *PCIF1*: GATCCGTTTGACGTACTCCA or ACTTAACATATCCTGCGGGG; non-targeting controls: ACGGAGGCTAAGCGTCGCAA or CGCTTCCGCGGCCCGTTCAA (Control1 and Control2 respectively, selected from the human GeCKO v2 CRISPR screening library). Annealed double-stranded DNA oligonucleotides corresponding to the gRNAs were ligated into a piggybac vector engineered to contain a U6-gRNA cassette in addition to a CMV promoter driving Cas9-T2A-eGFP. Plasmids were transfected into K562 cells using LipoD293 (SignaGen, Cat. # SL100668), according to the manufacturer’s instructions. The transfected cells were sorted twice by flow cytometry based on eGFP expression. Loss of METTL3 or PCIF1 protein expression was confirmed by western blotting using anti-METTL3 and PCIF1 antibodies (Bethyl Lab. Cat. #A301-567A, #A304-711A).

#### LC-MS/MS analysis

For the detection and quantification of m^6^A and cap-adjacent m^6^Am in 2x polyA+ RNA, 400 ng of 2x polyA+ RNA was decapped using 25 Units of RppH (NEB, Cat. #M0356S) in 1X ThermoPol buffer (NEB, Cat. #B9004S) for 3 hours at 37°C, followed by clean up with Zymo RNA Clean and Concentrator-5 Kit (Cat. #R1013). Subsequently, decapped RNA was digested to nucleotides using 20 units of Nuclease P1 (NEB, Cat. #M0660) in a buffer containing 50 mM sodium acetate (pH 5.5) and 0.05 μM 2’-deoxyadenosine (internal standard) for 3 hours at 37°C. Nucleotides were then dephosphorylated to nucleosides by the addition of 2 units of Shrimp Alkaline Phosphatase (NEB, Cat. #M3071S) in 1X CutSmart buffer for 1 hour at 37°C. After digestion, the sample volume was brought to 100 μL with ddH2O followed by filtration using YM-10 centrifugal spin column (Millipore, Cat. #MRCPRT010). 5 μL of the filtered solution was analyzed by LC-MS/MS.

The separation of nucleosides was performed using an Agilent 1290 Infinity HPLC system with an Agilent XDB-C18 reversed-phase column (4.6 x 150 mm, 5 μm). The mobile phase A was water with 0.1% (v/v) formic acid and mobile phase B was methanol with 0.1% (v/v) formic acid. Online mass spectrometry detection was performed using an Agilent 6460 triple quadrupole mass spectrometer in positive electrospray ionization mode. Quantification of each nucleoside was accomplished in dynamic multiple reaction monitoring (dMRM) mode by monitoring the transitions of 268/136 (A), 252/136 (dA), 282/150 (m^6^A), 296/150 (m^6^Am). The amounts of A, dA, m^6^A and m^6^Am in the samples were quantified using corresponding calibration curves generated with pure standards.

#### Nanowell and flow cell scanning

To identify cell types or quantify cell surface protein expression levels on the nanowell array (Figure 2A), cell preparation and imaging were performed as previously described with some modifications (Gierahn et al., 2017). K562 and YAC1 cells were resuspended in F-PBS buffer (1X cold PBS with 2% FBS) and nuclear staining dyes (1:1000 Hoechst 33342 and SYTO9 or SYTO87; Invitrogen, Cat. #62249, #S34854, #S11363). Cells were washed twice with and resuspended in F-PBS. The nanowell array was washed with 6 mL of 95% ethanol once and 1X cold PBS, 5 times. To maximize the number of single-cell occupied wells, 3.0 x 10^4^ cells (∼60% of the number of nanowells) of K562 and YAC1 mixture (1:1 ratio) were loaded onto the array and washed twice with 6 ml of 1X cold PBS. The array was imaged with a Zeiss (LSM 800) fluorescent microscope with a 5X objective. MUTZ3 cells were resuspended in F-PBS buffer with 1:100 anti-CD14 (Beckman Coulter, Cat. #IM0643) and anti-CD34 (BD Biosciences, Cat. #348050) primary antibodies for 30 min at room temperature and washed twice with F-PBS buffer. The MUTZ3 cells were stained in F-PBS buffer with 1:200 fluorophore-labeled anti-mouse IgG1 and anti-mouse IgG2a (ThermoFisher, Cat. #A21123 and #A21241) cross-adsorbed secondary antibodies and 1:1000 Hoechst 33342 for 30 min at room temperature.

After nanowell scanning for cell type identification, the array then hybridized to the passivated poly(dT50) coverslip. To lyse the loaded cells, the array was covered with 400 μL lysis buffer (20 mM Tris-HCl, pH8.0, 150 mM NaCl, 5 mM MgCl_2_, 1 mM EDTA, 0.2% NP-40, SUPERase·In RNase Inhibitor) and immediately sealed with the poly(dT50) coverslip using the manual clamp (Agilent, Cat. #G2534A). The sealed array was incubated for 1 h at 4°C and then submerged in 1X cold PBS to detach the coverslip. The custom Secure Seal Flowcell was assembled with the coverslip and washed three times with 1X SSC/ 0.05% SDS. FluoSphere beads (ThermoFischer, Cat. #F8807) were applied on the flow cell as an alignment marker through all rounds of antibody detection and serial hybridization for seqFISH. To visualize the transcriptome spatially separated on the coverslip from each nanowell, the captured RNA was labeled with 100 nM Cy3-dATP by Klenow exo- (NEB, Cat. #M0212) according to the manufacturer’s instructions, and 31 x 51 field of views (FOVs) were imaged using a customized TIRF microscope.

#### Primary probe design for seqFISH

Gene-specific primary probes were designed as previously described with some modifications (Eng et al., 2017). Probe sets were crafted separately for each gene using OligoMiner (Beliveau et al., 2018) and then refined as a full set to mitigate cross-hybridization in the experiment. Individual probe sets were first crafted using exons only from the consensus regions of all spliced isoforms of the gene, filtered by ClustalW. We chose 25-30 nt sequences corresponding to such exons and calculated their GC content. Probe sequences that fell outside of the allowed GC range (45-70% in this case) were excluded. We also removed any probe sequences that contained five or more consecutive nucleotide bases of the same kind. All probes were at least 2-nt distance from each other on the target sequence. To minimize cross-hybridization between probe sets, a local BLAST database was constructed from all the viable probe sequences, and the probes were queried against it. All probes with matches of 17 nt or longer between probes were removed by dropping the matched probe from the probe set.

For this experiment, the targeted probe set size range was set to 20–30 probes. Any probe set with more than 32 probes was trimmed down by removing probes with the farthest distance from the targeted 55% GC content. We used the 20 nt readout sequences as previously described with some modifications (Eng et al., 2017). We used BLAST to remove any sequences that matched with any contiguous homology sequences longer than 14 nt to the human transcriptome. The reverse complements of these readout sequences were included in the primary probes, and we added ‘TA’ gap sequence between probe and readout sequences. Primary probes were ordered as OligoPools from Integrated DNA Technologies (IDT) and resuspended in the primary probe hybridization buffer composed of 2X SSC (ThermoFisher, Cat. #15557036), 30% formamide (Sigma, Cat. #F9037) and 10% (w/v) Dextran Sulfate (Sigma, Cat. #D8906).

#### Readout probe synthesis for seqFISH

20-nt, 5’-amine-modified readout probes (IDT) were resuspended in 100 mM Sodium Bicarbonate Buffer. Azido-PEG3-SS-NHS (Conju Probe, Cat. #CP-2060) was reacted with 5’ amine-modified oligonucleotides at a 1:100 molar ratio in Sodium Bicarbonate Buffer for at least 6 h or overnight at room temperature on a shaker. Then, the crude mixture was purified using Illustra NAP-5 columns (GE Healthcare, Cat. #17-0853-01) and stored at -20°C. The oligonucleotides were mixed with AFDye 647 DBCO (Click Chemistry Tools, Cat. #1302-1) at a 1:10 molar ratio in Sodium Bicarbonate Buffer for at least 2 h at room temperature and added to Azide Magnetic Beads (Click Chemistry Tools, Cat. #1036-1) at a 1:20 molar ratio for 4 h at room temperature on a shaker. To remove the magnetic beads, the mixtures were placed on a magnet and the supernatant containing dye-labeled cleavable oligonucleotides was removed and stored at -20°C until the seqFISH experiment.

#### Gene expression measurement using seqFISH

Sequential fluorescence *in situ* hybridization was performed as previously described with some modifications (Eng et al., 2017). Once poly(A)+ RNA and m^6^A-modified RNA on the poly(dT) coverslip were imaged, the surface was treated with 40 U/mL proteinase K in 100 μL T50 buffer for 15 min at 37°C to completely clear antibody probes. A mixture of 222 probes (1 nM/probe) in 100 μL hybridization buffer containing 2X SSC (ThermoFischer, Cat. #15557036), 30% formamide (Sigma, Cat. #F9037), 10% (w/v) Dextran Sulfate (Sigma, Cat. #D8906), and 200 U/mL SUPERase·In RNase Inhibitor was hybridized to the target mRNA at 37°C for at least 16 h in a humid hybridization chamber. After hybridization, the sample was washed for 30 min at room temperature with the washing buffer containing 2X SSC, 40% formamide, 0.1% Triton X-100, and 200 U/mL SUPERase·In RNase Inhibitor to eliminate nonspecific binding of the primary probes. The sample was then washed three times with 2X SSC and 200 U/mL SUPERase·In RNase Inhibitor. Each readout probe hybridization solution contained each dye-labeled readout oligonucleotide probe (10 nM) in the hybridization buffer comprising 2X SSC, 10% formamide, 10% (w/v) Dextran Sulfate (Sigma, Cat. #D4911), and 200 U/mL SUPERase·In RNase Inhibitor. Each serial hybridization took 15 min at 37°C for optimal fluorescent signals followed by washing once for 2 min with a high-stringency buffer containing 2X SSC and 20% formamide. Once the readout probe hybridization was complete, FOVs were imaged with 500 ms exposure in oxygen-scavenging T50 buffer containing 10 mM Tris-HCl pH 8.0, 50 mM NaCl, 0.05% Tween-20, 1 mM Trolox, 50 nM Protocatechuate 3,4-Dioxygenase, 1 mg/mL protocatechuate, and 200 U/mL SUPERase·In RNase Inhibitor. Imaging was done using customized TIRF microscopy. Once imaging was complete, a reduction buffer containing 2X SSC, 50 mM TCEP (Sigma, Cat. #646547), 0.1% Triton X-100, and 200 U/mL SUPERase·In RNase Inhibitor was flowed into the flow cell and incubated for 2 min to completely cleave fluorophores on the readout oligonucleotides. Then, 2X SSC buffer supplemented with 200 U/mL SUPERase·In RNase Inhibitor was flown through the flow cell repeatedly 5 times for 2 min to remove the remaining TCEP solution. The whole process was repeated for each gene of interest, until 9 rounds of hybridizations were imaged. Generally, a seqFISH experiment takes ∼7 h for imaging 200∼300 FOVs.

### Quantification and statistical analysis

#### Registration of scanning images

To find X/Y coordinates of and measure the fluorescence intensity of single cells on the multicolor images of the nanowell array, we used automated image analysis by ‘find maxima’ algorithm and custom scripts on ImageJ (https://github.com/Kim-KL/scRNAmod). Scatter plot analysis of fluorescence intensities was used to identify cell types. A cell occupancy matrix (COM, 121 x 381) was used to identify the nanowells occupied with a single cell. From 31 x 51 FOV images on the flow cell, automated image analysis by custom scripts on ImageJ and R was used to count the number of fluorescence signals as a quantity of captured RNA molecules and generate the matrix of RNA density per FOV (RDM, 31 x 51). The RDM was registered in the COM by searching the 31 x 51 subset of COM with the highest 2D correlation coefficient using 2D cross correlation in R (Figures 2A and S2C–S2E) (Brown, 1992). With reference to the registered COM subset, doublets and empty wells were excluded from further analysis, and each registered single cell was linked with cell phenotypes from multicolor fluorescence images on the nanowell array.

#### Single-molecule image processing

Image processing and analysis were performed as previously described with some modifications (Eng et al., 2017; Kim et al., 2018). To remove background signal, the rolling-ball background subtraction with a radius of 3 pixels and Gaussian blur with 1.1 sigma value on ImageJ were applied on the single-molecule images. All fluorescent signals that could be identified as potential RNA or modification signals were found by finding local maxima in the image above a predetermined pixel threshold in the FOVs and fitting to a 2D Gaussian function to determine the center positions of the molecules to sub-pixel accuracy. The spots in different color channels, whose center was within a distance of 1 pixel (∼100 nm), were determined as colocalization signals using a custom Python script (https://github.com/Kim-KL/scRNAmod).

As the bright fluorescent signals from the FluoSphere beads (660/680) permanently appeared in the FOV, these signals were used to align all sets of images including antibody detection and seqFISH using a phase cross correlation.

#### Unsupervised mapping of cell types from seqFISH

To visualize similarities between single cells in two-dimensional space, we employed t-distributed stochastic neighbor embedding (t-SNE). We processed seqFISH images into a expression matrix consisting of 9 genes and 111 single cells. This matrix was annotated by cell color intensity, captured RNA density, and m^6^A-modified RNA density for single cells. Gene expression values were normalized by dividing gene-specific counts with the total number of captured RNA molecules for each cell. We then computed log_2_ transformed expression values, followed by subtraction of the average gene expression value across all cells. Single cell housekeeping gene expression values (GAPDH, ENO1, EEF2) were used as a quality control. For t-SNE visualization, we used the Rtsne implementation in R and default parameters, except setting the perplexity to 10. The visualization was used to highlight additional cell parameters, such as cell color intensity profile, capture RNA density, m^6^A level, and gene expression levels.

## Supplemental information

Document S1. Figures S1–S3

Table S1. Direct RNA sequencing and m6A detection at single-molecule resolution, related to Figure 1

Table S2. seqFISH probe sequences, related to Figure 3

Table S3. Plasmids for CRISPR KO cell lines, related to Figure S1 and STAR Methods

Document S2. Article plus supplemental information
