# Supplementary material for: Systematic detection of m6A-modified transcripts at single-molecule and single-cell resolution
Source: Cell Rep Methods. 2021 Aug 2;1(5):100061. doi: 10.1016/j.crmeth.2021.100061 (PMC8562683; doi:10.1016/j.crmeth.2021.100061)
Supplement: Document S1. Figures S1–S3 [file mmc1.pdf]

Cell Reports Methods, Volume *1*

Supplemental information

Systematic detection of m^{6}A-modified transcripts at single-molecule and single-cell resolution

Kyung Lock Kim, Peter van Galen, Volker Hovestadt, Gilbert J. Rahme, Ekaterina N. Andreishcheva, Abhijeet Shinde, Elizabeth Gaskell, Daniel R. Jones, Efrat Shema, and Bradley E. Bernstein

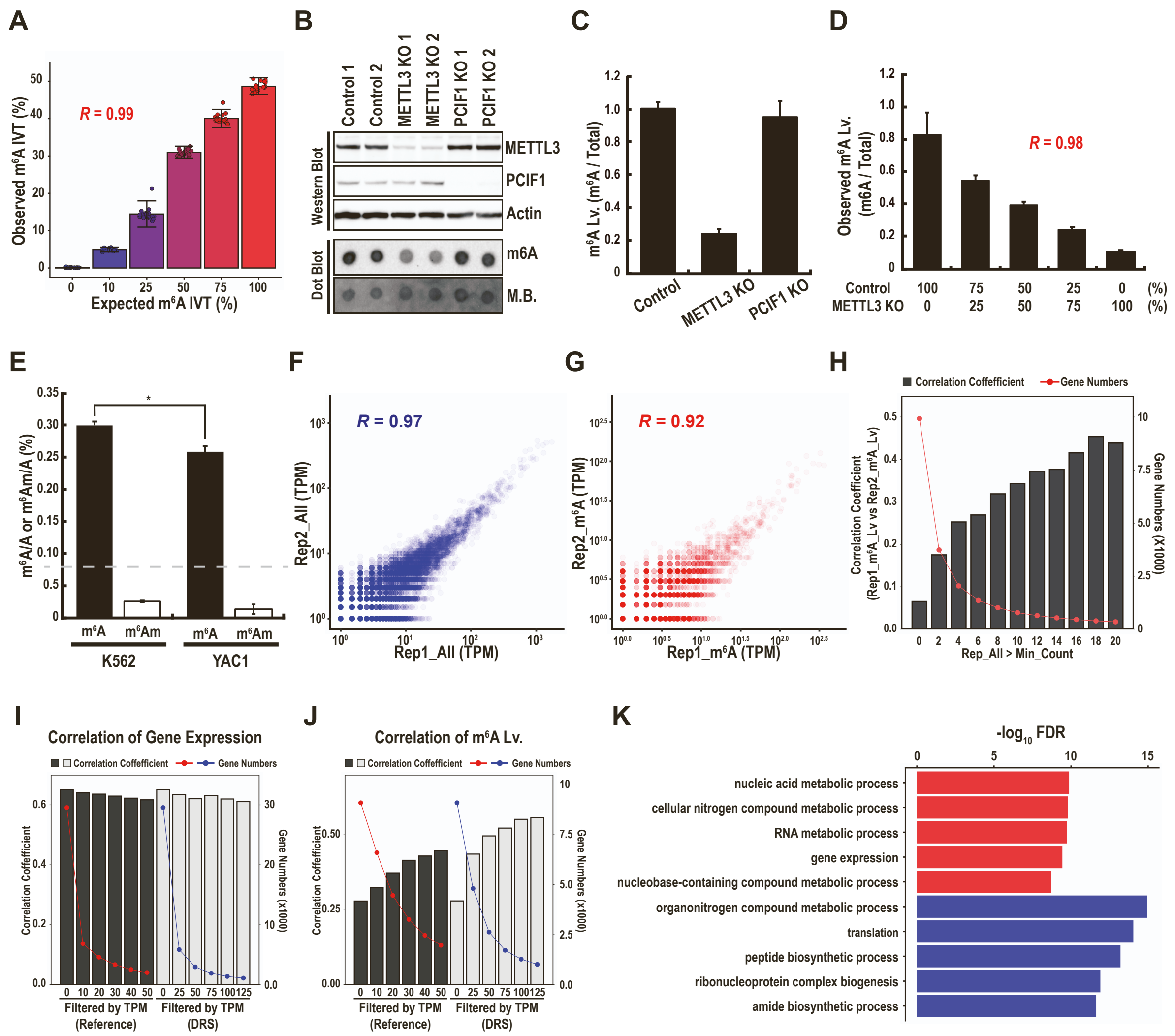

Figure S1. Validation of antibody detection for m^{6}A RNAs and gene expression profiling at single-molecule resolution, Related to Figure 1. A. Bar plot compares the level of observed versus expected m^{6}A levels of m^{6}A(-/+) RNAs in 6 independent single-molecule assays. The methylated RNA transcripts were generated via *in vitro* transcription utilizing N6-methyladenosine-5'-triphosphate. The m^{6}A-positive RNAs were mixed with unmodified RNA molecules at the indicated ratios to produce specific m^{6}A levels. The Pearson correlation coefficient and *P*-value are 0.99 and 1.1 x 10^{-4}, respectively (n = 10). B. Top: CRISPR-mediated *METTL3* or *PCIF1* knockout (KO) in K562 cells was assessed by anti-METTL3 and PCIF1 immunoblotting. β-actin, loading control. Bottom: Anti-m^{6}A dot blot using 100 ng of double polyA-selected (2x polyA+) RNA from wild-type (WT), *METTL3* or *PCIF1* KO K562 cells. C. Bar plot representing m^{6}A quantifications of 2x polyA+ RNAs of WT, *METTL3* or *PCIF1* KO K562 cells in single-molecule assays. D. Bar plot shows the linearity of m^{6}A detection rates in 5 independent single-molecule assays using a mixture of 2x polyA+ RNAs

of WT and *METTL3* KO K562 cells at the indicated ratios. The Pearson correlation coefficient and *P*-value are 0.98 and 1.8 x 10^{-3}, respectively (n = 2). E. Liquid chromatography-MS/MS m^6A or m^6Am to A percentages of 2x polyA+ RNAs from K562 and YAC1 cells. Dashed line: lower limit of the calibrated range per our m^6Am standard inputs. *$p < 0.05$, error bar represents standard error (n = 2). (F, G) Scatter plot across (F) all transcripts and (G) m^6A-positive transcripts using LQ-DGE. H. Bar and line plots show correlation coefficient between the replicates of our modified LQ-DGE assay and qualified gene numbers, respectively, depending on the cut-off of expression levels as read counts. (I, J) Bar plots show the correlation coefficients of gene expression (I) and m^6A levels (J) between our modified LQ-DGE (total: 0.51 M reads and m^6A(+): 0.14 M reads) and m^6A-LAIC-seq (m^6A-negative or m^6A-positive sample: each 50 M reads). Line plots show qualified gene numbers depending on the cut-off of gene expression levels. K. Gene-Ontology (GO) analysis of top and bottom 500 genes with the highest (red) and lowest (blue) methylation levels.

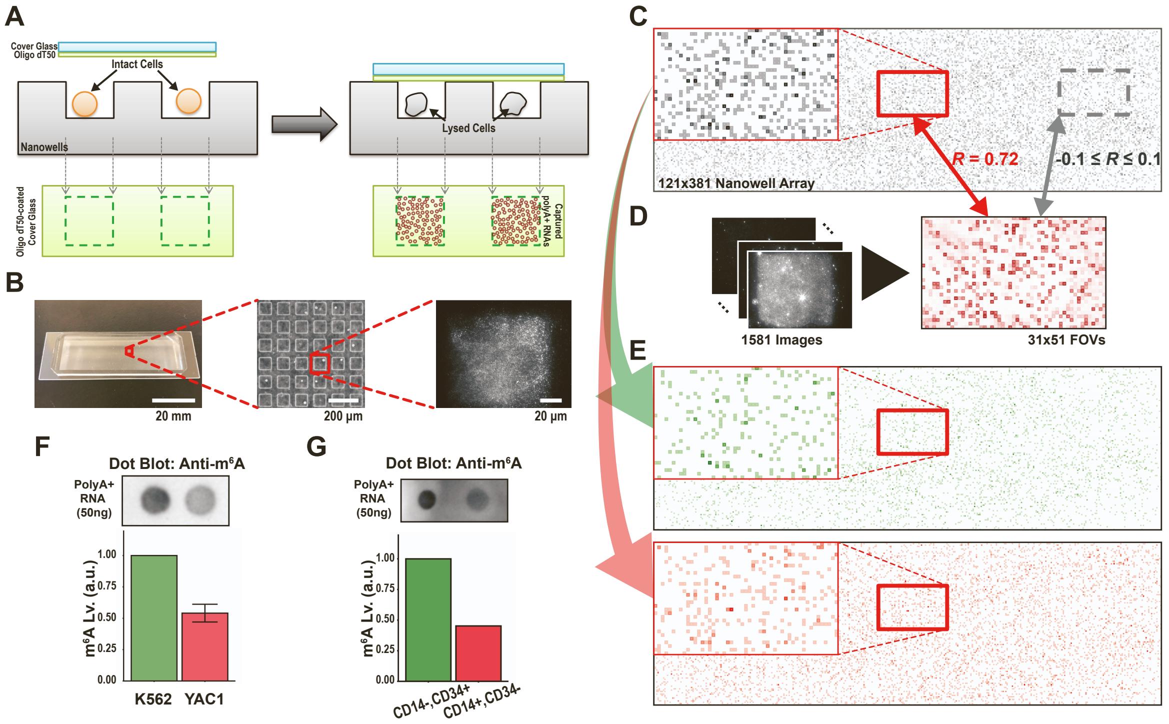

Figure S2. Single cell analysis using a nanowell array, Related to Figure 2. A. Spatial isolation of single cells and their transcriptomes. Single cells were captured randomly and lysed on a nanowell array. After lysis, the array was immediately sealed with an oligo dT-coated coverslip. B. Cell-loaded nanowells, optimized Poisson loading of single cells on nanowell array, and cluster of polyA+ RNAs on an oligo dT-coated coverslip. C. Cell occupancy matrix (COM) from a 46,101 nanowell capture array (121x381 nanowells). The number of cells per nanowell was determined through on-array fluorescent imaging of stained nuclei. Based on the 2D image pixel coordinates of nuclei, each cell was assigned to a specific nanowell. D. RNA density matrix (RDM) from a selection of 1,581 nanowell images (31x51 FOV) representing the number of captured polyA+ RNA molecules per FOV. Single cell position registration was performed using a custom R script and 2D cross-correlation to overlay the COM and RDM matrices. E. COM of K562 (green) or YAC1 (red) annotated with color intensity profile of single cells from on-array multicolor fluorescent images. (F, G) Anti-m^{6}A dot blot using 50 ng RNA that was polyA-selected twice using Oligo dT beads and quantification of the dot blot result by densitometry analysis. F. K562 and YAC1. G. subpopulations of MUTZ3 cells sorted by flow cytometry.

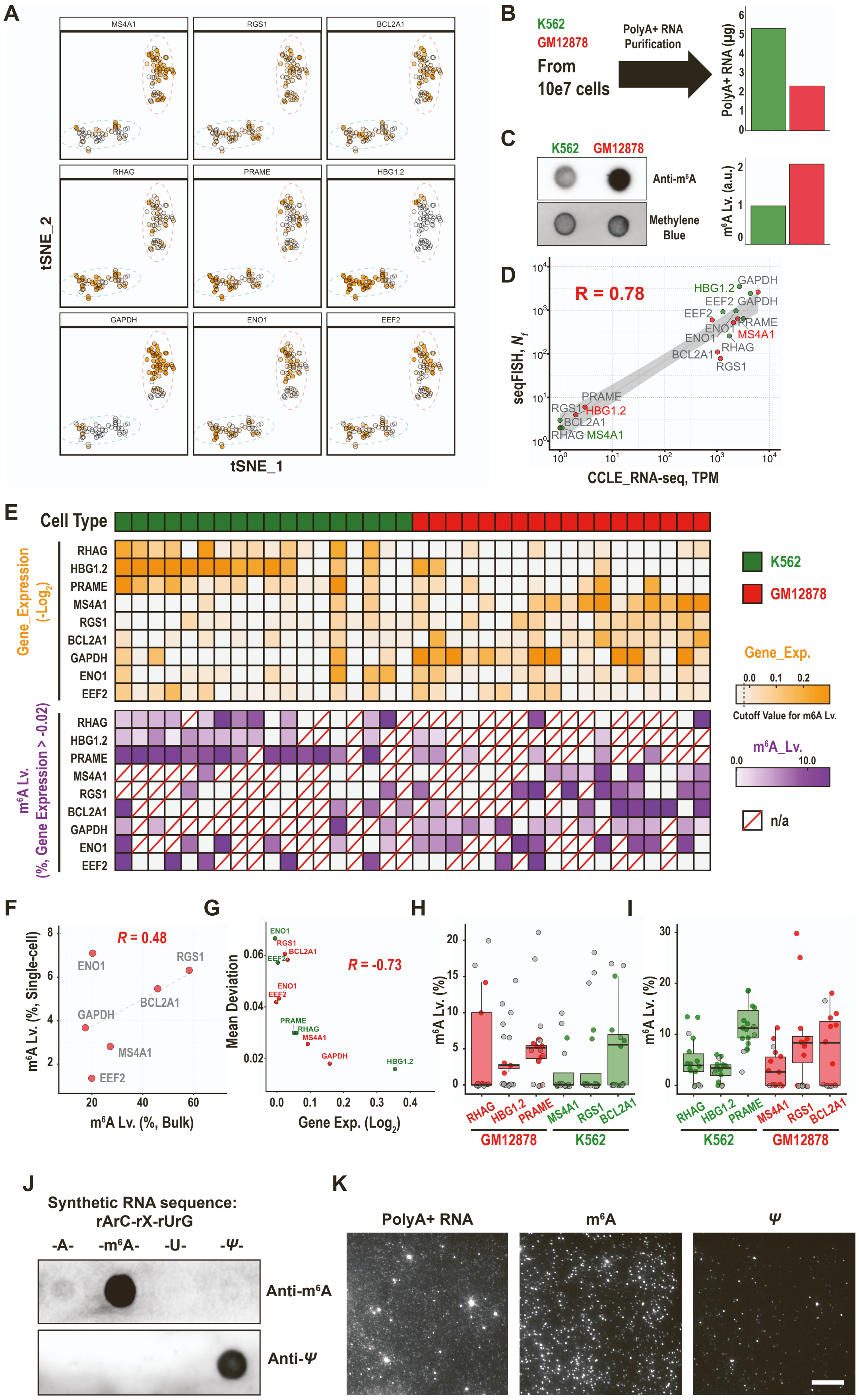

A
B
K562
GM12878
From
10e7 cells
PolyA+ RNA
Purification
PolyA+ RNA (μg)
C
K562
GM12878
Anti-m6A
Methylene
Blue
m6A Lv. (a.u.)
D
R = 0.78
seqFISH, Nf
CCLE_RNA-seq, TPM
tSNE_2
tSNE_1
E
Cell Type
Gene_Expression (-Log2)
m6A Lv. (%, Gene Expression > -0.02)
RHAG
HBG1.2
PRAME
MS4A1
RGS1
BCL2A1
GAPDH
ENO1
EEF2
K562
GM12878
Gene_Exp.
Cutoff Value for m6A Lv.
m6A_Lv.
n/a
F
R = 0.48
m6A Lv. (%, Single-cell)
m6A Lv. (%, Bulk)
G
R = -0.73
Mean Deviation
Gene Exp. (Log2)
H
I
m6A Lv. (%)
J
Synthetic RNA sequence:
rArC-rX-rUrG
-A-
-m6A-
-U-
-Ψ-
Anti-m6A
Anti-Ψ
K
PolyA+ RNA
m6A
Ψ

Figure S3. Combination of single-cell analysis and seqFISH, Related to Figure 3. A. t-distributed stochastic neighbor embedding (t-SNE) visualization of 111 qualified single-cell seqFISH profiles colored with relative expression levels of 9 genes (orange). B. Quantification of 2x polyA+ RNA purified from 10 million K562 (green) or GM12878 (red) cells using oligo dT beads. C. Left: Anti-m^6A dot blot using 50 ng polyA+ RNA of indicated cell lines. Right: Quantification of the dot blot result by densitometry analysis. D. Scatter plot showing correlation between bulk seqFISH results and CCLE RNA-seq data (K562, green; GM12878, red). N_f, average number of fluorescent molecules per imaging area (15,400 μm^2). E. Heatmap showing relative expression of 9 genes (orange) and gene-specific m^6A levels (purple) across single cells (K562, green; GM12878, red). m^6A levels of genes with log-mean expression greater than cutoff value (-0.02) are shown. Positions marked with a red diagonal line represent 'Not Applicable' values (n/a), meaning that the single-cell gene expression is not greater than cutoff value. F. Scatter plot representing the correlation between gene-specific m^6A levels of GM12878 at the single-cell level and in the bulk cell line experiment (Figure 1). G. Scatter plot showing the correlation between relative gene expressions and mean deviation of m^6A levels implying the variable volatility. The Pearson correlation coefficient is indicated on the plot. H-I. Box and column scatter plots show single-cell gene-specific m^6A levels with log-mean gene expressions greater than -0.02 (K562, green; GM12878, red). Gray dots in the column scatter plots represent gene expression measurements with 'n/a' m^6A levels. J. Anti-m^6A and anti-pseudouridine (Ψ) dot blot assays using 50 ng synthetic RNA fragments. K. Iterative single-molecule blotting assay to identify combinatorially modified RNA molecules. From GM12878 lysates, polyA+ RNAs were captured on the oligo dT-coated flow cell. The captured RNAs were labeled with Cy3-dATP using a Klenow exo- reaction. The m^6A-modified RNAs were detected by 0.33 nM anti-m^6A antibody coupled with 1.33 nM Alexa Fluor 647-labeled secondary antibody. The antibody complexes were cleared by Proteinase K, followed by detection of Ψ-modified RNAs using 0.33 nM anti-Ψ antibody and the secondary antibody. Scale bar, 10 μm.
